# Clinical and Practical Implications of Storage Media used for Tooth Avulsion

**DOI:** 10.5005/jp-journals-10005-1427

**Published:** 2017-06-01

**Authors:** Vineet IS Khinda, Gurpreet Kaur, Gurlal S Brar, Shiminder Kallar, Heena Khurana

**Affiliations:** 1 Professor and Head, Department of Pedodontics and Preventive Dentistry, Genesis Institute of Dental Sciences & Research, Ferozepur, Punjab India; 2 Postgraduate Student, Department of Pedodontics and Preventive Dentistry, Genesis Institute of Dental Sciences & Research, Ferozepur, Punjab India; 3 Reader, Department of Pedodontics and Preventive Dentistry, Genesis Institute of Dental Sciences & Research, Ferozepur, Punjab India; 4 Reader, Department of Pedodontics and Preventive Dentistry, Genesis Institute of Dental Sciences & Research, Ferozepur, Punjab India; 5 Senior Lecturer, Department of Pedodontics and Preventive Dentistry, Genesis Institute of Dental Sciences & Research, Ferozepur, Punjab India

**Keywords:** Extra-alveolar storage period, Osmolarity, Periodontal ligament cells, pH, Storage media.

## Abstract

Replantation is being widely accepted as an effective treatment option for an avulsed tooth. However, the long-term fate of replanted teeth is unpredictable; it is dependent on various factors, such as the time interval between avulsion and replantation, extra-alveolar storage period (dry storage or storage media), the vitality status of pulp or periodontal tissues and the type and period of splinting. The appropriate use of storage media is an important clinical factor affecting the postoperative prognosis of avulsed teeth following replantation. Hank’s balanced salt solution and pasteurized milk are considered to be the most appropriate and clinically recommended storage media for avulsed teeth. The present review discusses the various available storage media for avulsed teeth and their potential maintenance of the vitality of periodontal ligament cells. A brief overview of the effect of clinical factors, such as the storage time, pH, and the osmolar-ity of storage media on their efficacy is included.

**How to cite this article:**

Khinda VIS, Kaur G, Brar GS, Kallar S, Khurana H. Clinical and Practical Implications of Storage Media used for Tooth Avulsion. Int J Clin Pediatr Dent 2017; 10(2): 158-165.

## INTRODUCTION

Tooth avulsion is a complex traumatic injury characterized by the complete dislodgement of the tooth from its socket, which causes severe damage to the supporting tissues, vascular and nerve structures, which requires a prompt and correct emergency management for the good prognosis.^1^ When an injury occurs, immediate replantation is the recommended treatment for an avulsed permanent tooth to prevent any further injury to the periodontal ligament (PDL) in order to minimize the risk of post-replantation resorption of either an inflammatory or a replacement nature.^2^

The prognosis of a replanted tooth depends on the viability of the PDL cells remaining on the root surface, integrity of root cementum, and minimal bacterial contamination, which are directly related to the extra-alveolar time, type of storage after avulsion, and root surface alterations.^3^ Immediate replantation of avulsed teeth impacts positively on the viability of PDL cells and results in PDL healing in up to 85% of mature teeth. Replantation of a tooth within 5 minutes usually ensures prompt return of the PDL cells to normal function. However, after more than 15 minutes of dry storage, the precursor, progenitor or stem cells are no longer able to differentiate into fibroblasts. After 30 minutes of dry storage, virtually all of the PDL cells remaining on the tooth root are likely to have become necrotic.^4^ However, immediate reimplantation rarely occurs due to several factors, such as stress, lack of knowledge, etc. Thus, proper storage of the avulsed tooth is necessary to prevent PDL necrosis.

## EFFECT OF STORAGE MEDIA ON PERIODONTAL HEALING

Teeth are usually subjected to a period of desiccation between their avulsion and replantation. Therefore, it is desirable to replant the avulsed tooth as quickly as possible to ensure the maximal viability of PDL cells (fibroblasts) attached to the root surface. As dry storage is detrimental to the preservation of the PDL, the avulsed tooth must be prevented from drying by the use of storage media of ideal osmolality and pH. A number of clinical studies have indicated a dependence of periodontal and pulpal healing on storage period and media. Therefore, in cases where an immediate replantation is not feasible, use of a storage medium is prudent to enhance and preserve the vitality of PDL fibroblasts of an avulsed tooth. In a clinical setup, a medium should possess certain properties to make it an acceptable storage medium for avulsed teeth.^5^

## IDEAL REQUIREMENTS OF A STORAGE MEDIUM FOR AVULSED TEETH

Ideal storage medium should:

- Have antimicrobial characteristics.
- Be capable of preserving the feasibility of cellular PDL.
- Be able to maintain the viability of periodontal fibers for an acceptable period of time.
- Favor proliferative capacity of cells and should have the same osmolality as that of body fluids.
- Not react with body fluids.
- Not produce any antigen antibody reactions.
- Reduce the risk of post-reimplantation root resorption or ankylosis.
- Have a good shelf life.
- Effective in different climate and under different conditions.
- Wash off extraneous materials and toxic waste products.
- Aid in the reconstitution of depleted cellular metabolites.^5^

The present review summarizes the role of various storage media in periodontal healing in avulsed teeth and the ongoing developments in this field.

## VARIOUS TYPES OF STORAGE MEDIA USED FOR AVULSED TEETH

There are many solutions that have been proposed and/or tested as storage media for avulsed teeth. The following were identified and reviewed as a result of the literature search: Hank’s balanced salt solution (HBSS), Eagle’s medium (EM), milk, ViaSpan, Gatorade, propolis, tooth rescue box (Dentosafe), conditioned medium, contact lens solution, tap water, egg white, saliva, normal saline, ORS, and coconut water.^4^ The search for a single, ideal storage medium, i.e, capable of maintaining PDL and pulp cell viability, while presenting clonogenic capacity, antioxidant property, no or minimal microbial contamination, compatible physiological pH and osmolality, high availability, ready accessibility, and low cost is one of the main interests of dental trauma research.

## SALINE SOLUTION

Normal saline is a solution of 0.90% w/v of NaCl and osmolality of 280 mOsm/kg and despite being compatible to the cells of the PDL, it lacks essential nutrients, such as magnesium, calcium, and glucose, which are fundamental to the normal metabolic needs of the cells of the PDL.^2^ Moreira-Neto et al and Pileggi et al evaluated the viability of cultured cells and found 55% of living cells after 4 hours storage and 20% of mortality of cells after 45 minutes storage.^6^ Hence, normal saline appears to be suitable for short-term storage of avulsed teeth for about 2 hours, however, it is potentially damaging if the cells are stored for longer than this. Consequently, saline is not an adequate medium, however, it may be employed for short period of time, although, other storage media are not immediately available and when required for a short period of time.^2^

## SALIVA

Previous studies suggested that saliva can be used as an interim storage medium for avulsed teeth to prevent desiccation. According to Weine, patient’s own saliva is the best immediate transport medium for an avulsed tooth. It is also an immediately available storage medium at all the accident sites. However, more recent studies have indicated that saliva may not be the most suitable medium for extended (greater than 1 hour) storage of avulsed teeth.^5^ Saliva can be used as a storing medium for a short period of time, for it can damage the cells of the PDL, if used for longer than an hour. Its osmolality (60-70 mOsm/kg) is much lower than the physiologic, thus storage of avulsed teeth in saliva for 2 to 3 hours causes swelling and membrane damage of PDL cells and also the presence of microorganisms makes saliva a less desirable storage medium.^7^ In 1 hour, it can cause approximately twice as much damage as HBSS or milk. However, saliva storage produces one-third less cell damage than dry storage or storage in tap water. Thus, saliva can be considered to be an acceptable short-term storage medium (less than 30 minutes) and its use should be limited to cases where the extra-alveolar duration is less and other superior storage media are not available.^5^

## PASTEURIZED MILK

**Fig. 1:**
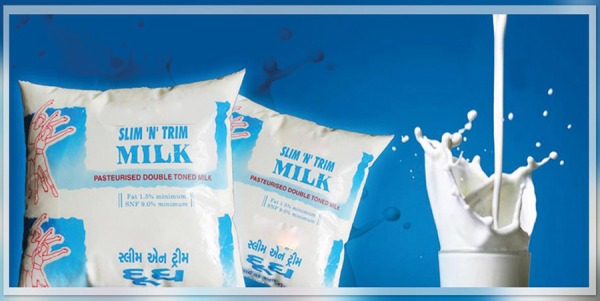
Pasteurized milk

Milk is significantly better than other solutions for its physiological properties, including pH (6.5-7.2) and osmolality (270 mOsm/kg) are compatible to those of the cells from the PDL; the easy way of obtaining it and for being free of bacteria, however, it is important that it is used in the first 20 minutes after avulsion. The favorable results of milk probably occur due to the presence of nutritional substances, such as amino acids, carbohydrates, and vitamins.^2^ Being a gland secretion, milk contains epithelial growth factor, which stimulates the proliferation and regeneration of epithelial cell rests of Malassez and activates the alveolar bone resorption. This will ultimately contribute to isolate the bone tissue from the tooth and decrease the likelihood of ankylosis. In spite of offering no conditions for the restoration of cell morphology, nor cell differentiation or mitosis, milk prevents cell death.^6^ Blomlof et al, Trope and Friedman recommend milk as an excellent storing solution for 6 hours, however, milk cannot revive the degenerated cells. Lekic et al demonstrated that milk was as effective as HBSS for storing avulsed teeth for up to 1 hour and superior to saline, saliva or water.^2^ At a cellular level, milk is ranked equal to HBSS as a storage medium although it loses its effectiveness after 2 hours. The osmolality of milk appears to be the most important factor. Milk has been widely recommended to dentists and general population for storing avulsed teeth to be replanted, being the second or third best transportation media for avulsed teeth (in order of preference), after ViaSpan and/or HBSS, according to the International Association of Dental Traumatology and the American Academy of Pediatric Dentistry due to its beneficial effects and characteristics and its ease of access at the moment of trauma (Fig. 1).^6^

## TAP WATER

Tap water has a pH of 7.4 to 7.79 and an osmolality of 30 mOsm/kg. It has inadequate characteristics to be used as storage medium for avulsed teeth because it has bacterial contamination, hypotonicity and nonphysi-ological pH, and osmolality, which favors the PDL cell lysis. Several studies have shown that cells stored in water did not maintain their morphology, with visible destruction and rapid cell death.^6^ Blomlof reported that water is damaging to PDL cells and is not a good storage medium at any time. Although, some studies have suggested that it may be accepted as a storage medium for very brief periods when there are no alternatives, however, it should be remembered that it is the least desirable storage medium available and its use will lead to ankylosis and replacement resorption.^4^ In view of this, tap water should be used only to avoid tooth dehydration, but it is inadequate for conservation of avulsed teeth.^6^

**Fig. 2:**
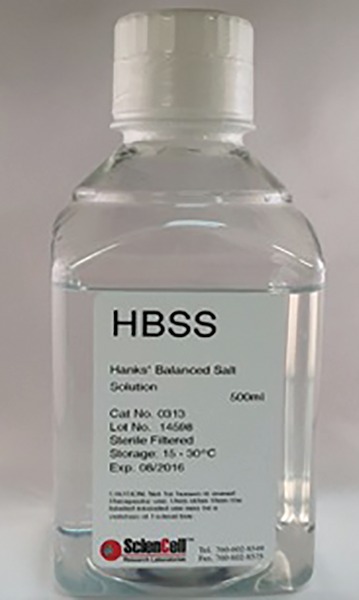
Hank’s balanced salt solution

## HANK’S BALANCED SALT SOLUTION (GIBCO® HBSS, Biological Industries, Life Tech)

**Fig. 3:**
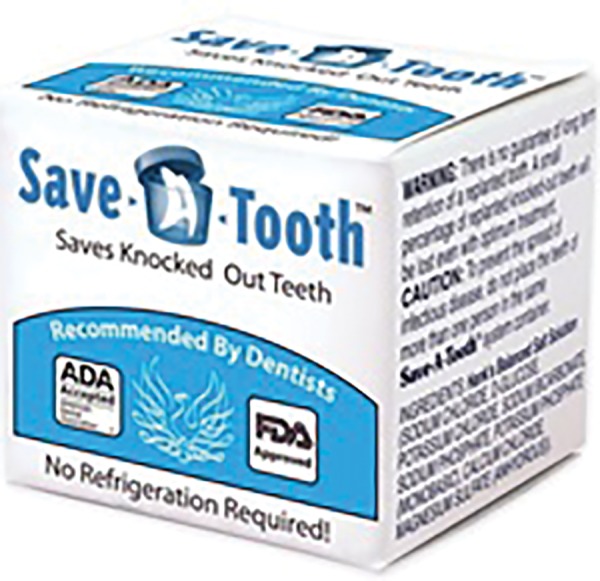
Save a tooth

The HBSS is a sterile, physiologically balanced isotonic standard salt solution, i.e., widely used in biomedical research to support the growth of many cell types.^5^ This solution is nontoxic; it is biocompatible with PDL cells, has pH balanced at 7.2, and has an osmolality of 320 mOsm/kg. It is composed of 8 g/L sodium chloride, 0.4 g/L of D-glucose, 0.4 g/L potassium chloride, 0.35 g/L sodium bicarbonate, 0.09 g/L sodium phosphate, 0.14 g/L potassium phosphate, 0.14 g/L calcium chloride, 0.1 g/L magnesium chloride, and 0.1 g/L magnesium sulphate.^7^ These ingredients can sustain and reconstitute the depleted cellular components of the PDL cells. Ashkenazi et al showed that HBSS was the most effective medium for preserving viability, mitogenicity, and clonogenic capacities of PDL cells for up to 24 hours at 4°C, when compared with culture medium (EM supplemented with 15% fetal calf serum and antibiotic solution containing 100 UI/mL penicillin, 50 μg/mL gentamicin, and 0.3 μg/mL fungizone), EM, milk, ViaSpan, and conditioned medium. Ashkenazi et al showed that the highest mitogenicity after 8 and 24 hours of storage was found in HBSS.^5^ The American Association of Endodontists recommends the use of HBSS as the storage medium of choice for treatment of avulsed teeth because of its ability to provide long-term preservation of PDL cell viability.^8^ However, HBSS is not available in most places where these traumatic events usually occur, such as in school, home, camps, and sports field settings, where people are physically active. Krasner and Person have developed an avulsed tooth storage system and emergency tooth preserving system (Save-A-Tooth), which contains HBSS, a net for holding the teeth atraumatically and a container for taking the submerged tooth to a clinician. The literature indicates HBSS to be superior to many other storage media in its ability to preserve cell vitality and viability. It is a desirable storage medium for avulsed teeth, even when the extra-alveolar period is extensive (between 72 and 96 hours) (Figs 2 and 3).^5^

**Fig. 4:**
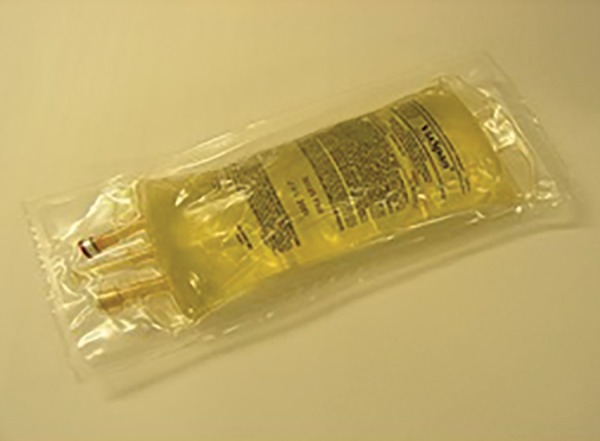
ViaSpan

## VIASPAN (DuPont Pharmaceuticals, Wilmington)

The ViaSpan is a cold transplant organ storage medium and it has been very effective for storing avulsed teeth. It has an osmolality of 320 mOsm/kg and its pH is around 7.4 at room temperature, which is ideal for the cellular growth.^7^ Ashkenazi et al have evaluated the effectiveness of 6 different storage media for avulsed teeth: Culture medium, milk, HBSS, ViaSpan, and conditioned medium for the observation of fibroblasts of the PDL. Interestingly, the clonogenic capacity of the stored cells kept in ViaSpan for 8 hours was high and comparable to HBSS and superior to milk. This capacity was diminished 65% after 24 hours when compared to the control and inferior to milk and HBSS. The biggest mitogenic capacity was found in the fibroblasts kept in milk or in HBSS and the lowest were found in conditioned medium or ViaSpan. Its high cost, short vitality expiration, and the difficulty in its availability, make it difficult to find and use this storage medium (Fig. 4).^2^

## EAGLE’S MEDIUM

**Fig. 5:**
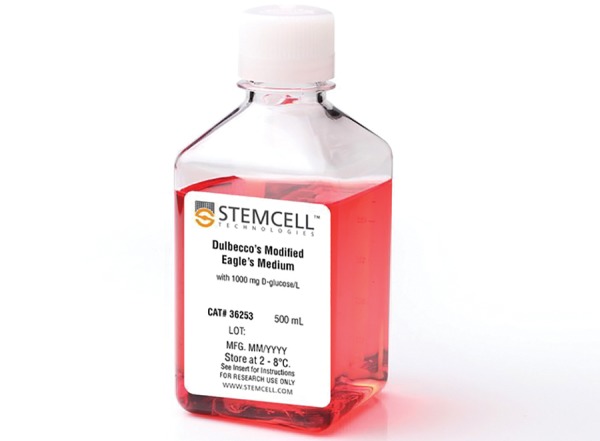
Eagle’s medium

Eagle’s minimal essential medium contains 4 mL of L-glutamine, 105 IU/L of penicillin, 100 μg/mL of streptomycin, 10 μg/mL of nystatin and calf serum (10% v/v).^7^ Ashkenazi et al observed that EM had relatively high viability, mitogenic and clonogenic capacity up to 8 hours of storage at 4°C. When the storage time was up to 24 hours, EM was less effective than milk or HBSS. The EM at 37°C is a recommended storage medium as it can preserve the PDL for extended periods before replantation. Thus, culture media possess a superior capacity to maintain the health of the PDL cells (Fig. 5).^2^

## GATORADE (Quaker Oats Company, Chicago, Illinois, USA)

**Fig. 6:**
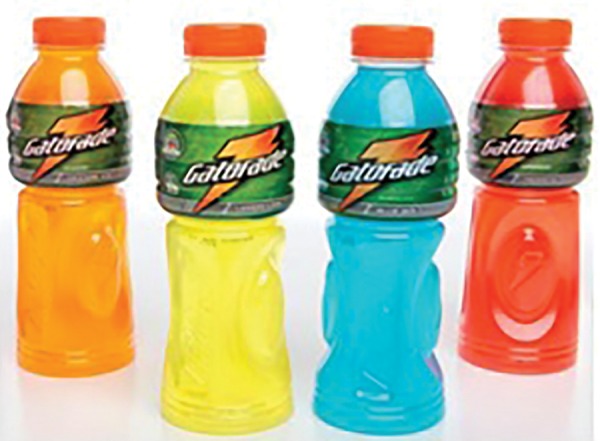
Gatorade

The oral rehydration fluid Gatorade is a potential transport medium, i.e., commonly found at sporting events. Gatorade is a noncarbonated sport drink often consumed by non-athletes as a snack beverage. It has a pH of 3 and osmolarity ranging from 280 to 360 mOsm/L.^5^ According to Harkacz et al, Gatorade did not turn to be an adequate storing medium for avulsed teeth due to its low pH and high osmolality.^7^ Gatorade preserves more viable cells than tap water but fewer than all other media, both at room temperature and on ice. Therefore, Gatorade can only serve as a storage medium if other more acceptable media are not available, rather than allowing the avulsed tooth to dry out (Fig. 6).^5^

## PROPOLIS

**Fig. 7:**
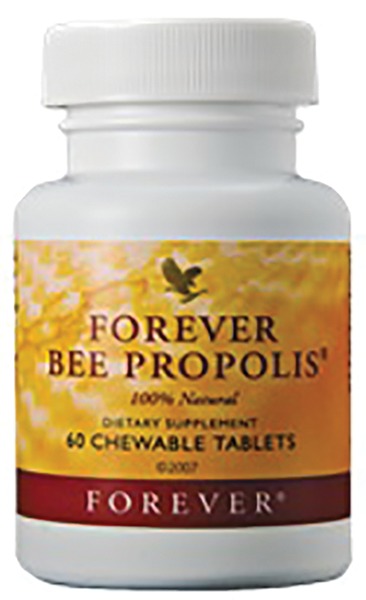
Propolis

Propolis has been a promising storage medium for the maintenance of cellular viability of the PDL of avulsed teeth. Propolis is a sticky resin that seeps from the buds or bark of trees, chiefly conifers. It consists of the following components: Resin (rich in flavonoids) (45-55%), waxes and fatty acids (23-35%), essential oils (10%), pollen proteins (5%), and other organic compounds and minerals. Propolis has antiseptic, antibiotic, antibacterial, antifungal, antiviral, antioxidant, anticarcinogenic, antithrombotic, and immunomodulatory properties.^5^ Margaret and Pileggi reported that teeth stored in propolis demonstrated the highest viability for PDL cells when compared with HBSS, milk, and saline.^9^ It can be considered as a favorable storing medium as it maintains cellular viability of the PDL, besides being antimicrobiotic, anti-inflammatory, and antioxidant (Fig. 7).

**Fig. 8:**
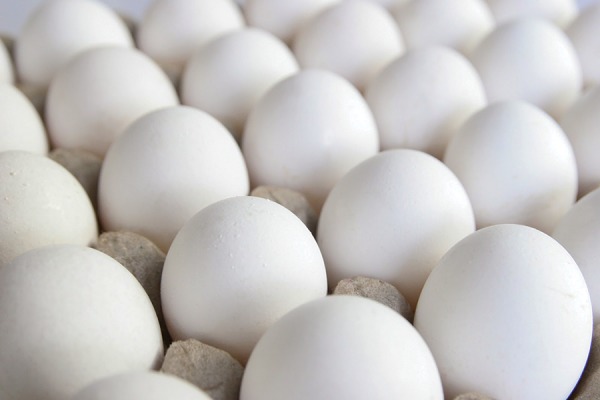
Egg white

**Fig. 9:**
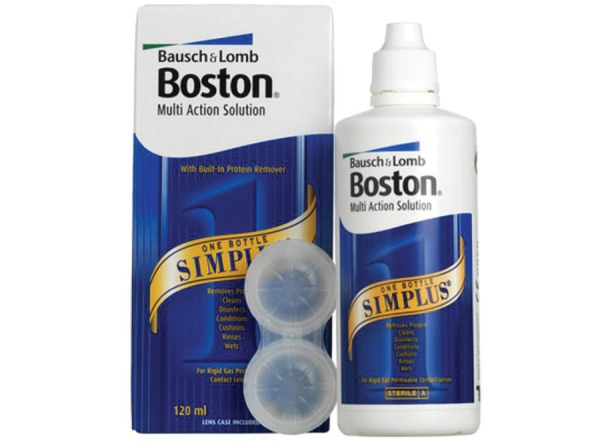
Contact lens solutions

## EGG WHITE

Egg white or egg albumin is considered as a good choice because of its high protein content, vitamins, water, lack of microbial contamination, and easy accessibility. It has a pH of 8.6 to 9.3 and its osmolality is 258 mOsmol/kg. It has shown better cell viability and significantly higher incident of PDL healing as compared to milk and equivalent cell viability as HBSS.^8^ Khademi et al^10^ compared milk and egg white as solutions for storing avulsed teeth and the results showed that teeth stored in egg white for 6 to 10 hours had a better incidence of repair than those stored in milk for the same amount of time (p < 0.05). Cellular growth occurs at an osmolality of 230 to 400 mOsm/kg and a pH of 6.6 to 7.8, however, its optimal growth happens at an osmolality of 290 to 300 mOsm/kg and pH of 7.2 to 7.4. However, no significant difference has been established between egg white and HBSS at storage times of 1, 2, 4, 8, and 12 hours, and egg white was more suitable than water and milk. It has been observed to be an excellent medium for up to 10 hours with the principle advantage being its availability (Fig. 8).

## CONTACT LENS SOLUTIONS

Contact lens solutions were initially thought to be of possible benefit as a storage solution for avulsed teeth because they are essentially saline solutions. They are comprised of a fatty acid monoester and a cationic antimicrobial component. Subsequent studies showed that the solutions for keeping contact lenses were worse than saline solution, milk, and HBSS. The presence of preservatives in its formula was harmful to the cells of the PDL and therefore, they are not recommended (Fig. 9).^7^

## TOOTH RESCUE BOX (DENTOSAFE)

The Dentosafe (Miradent, Germany) “tooth rescue box“ has been distributed to all schools in Austria and in parts of Germany and Switzerland. It contains a culture medium similar to the medium used during islet cell transplantation containing salts, amino acids, glucose, and vitamins. It has been shown to maintain the vitality of PDL cells for up to 48 hours at room temperature *in vitro.* At room temperature, the unopened box has a shelf life of 3 years.^4^ Pohl et al showed that all teeth placed in the Dentosafe solution shortly after avulsion healed with physiologic function and proposed that Dentosafe should be the standard equipment in first aid kits. Currently, this solution and device are not readily available in all countries.^11^

## COCONUT WATER

**Fig. 10:**
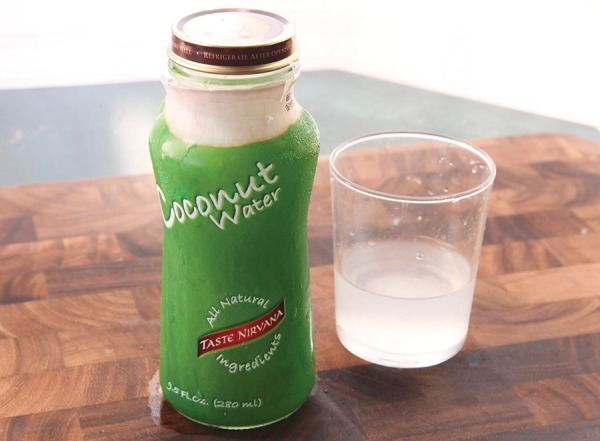
Coconut water

Coconut water is liquid endosperm of coconut; rich in amino acids, proteins, vitamins, and minerals.^12^ It is widely consumed to replace fluids, electrolytes (potassium, calcium, and magnesium), and sugar lost from the body. This natural isotonic fluid is available in its natural form directly from the coconut or in long shelf life packages and plastic bottles, mainly in tropical countries. Gopikrishna et al proposed coconut water as a promising medium for avulsed teeth and have shown it to be superior to HBSS, milk or propolis in maintaining the viability of PDL cells. As a result of its high osmolality, composition, and ready acceptance by the human body, coconut water has been studied as a potential interim storage medium for avulsed teeth. However, Moreira-Neto et al observed that coconut water has an acidic pH of 4.1, which is deleterious to cell metabolism and concluded that the capacity of the storage media in maintaining human fibroblast cell viability in a decreasing order was milk > saline and coconut water with sodium bicarbonate > coconut water > still mineral water.^13^ However, overall, the use of coconut water as a storage medium for avulsed teeth is not feasible under clinical conditions because of the difficulty of neutralizing the coconut water to obtain a pH of 7.0 (Fig. 10).

## ORAL REHYDRATION SALT-LIQUID

**Fig. 11:**
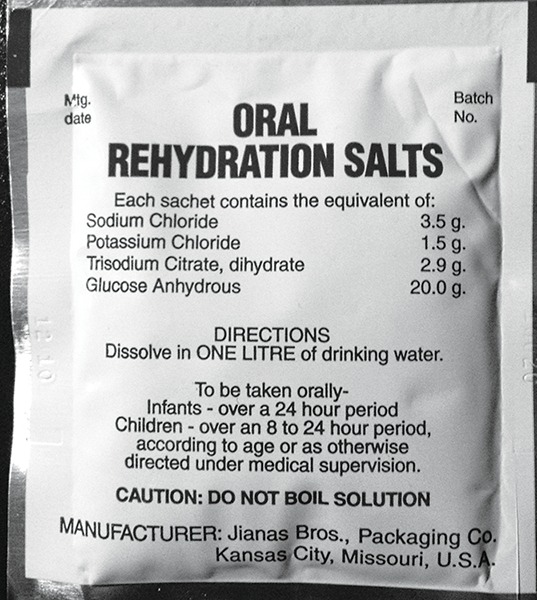
Oral rehydration salt-liquid

Subramaniam et al carried out a study to evaluate the efficacy of oral rehydration salt-liquid as a suitable medium for maintaining the PDL cell viability over different time periods and to compare its efficacy with that of two other storage media, HBSS and milk. They found oral rehydra-tion solution-liquid as storage medium to be as efficient as HBSS to maintain the viability of PDL cells, however, found it to be better than milk (Fig. 11).^14^

## GREEN TEA EXTRACT

**Fig. 12:**
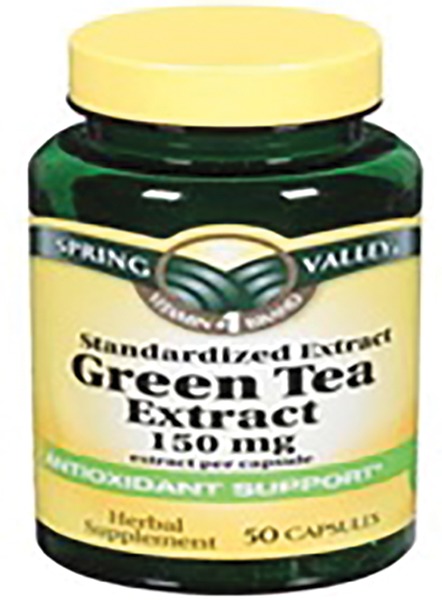
Green tea extract

Green tea extract (GTE) has been reported to have remarkable anti-inflammatory, antibacterial, and anticar-iogenic effects and to prolong allograft survivals. Hwang and Park investigated the efficacy of GTE as a storage medium for avulsed teeth and found that there was no difference in the PDL cell viability between GTE and HBSS medium, whereas GTE showed higher viability than milk, water, and commercial green tea. Therefore, GTE could be a suitable alternative storage medium for avulsed teeth (Fig. 12).^15^

## ASCORBIC ACID

**Fig. 13:**
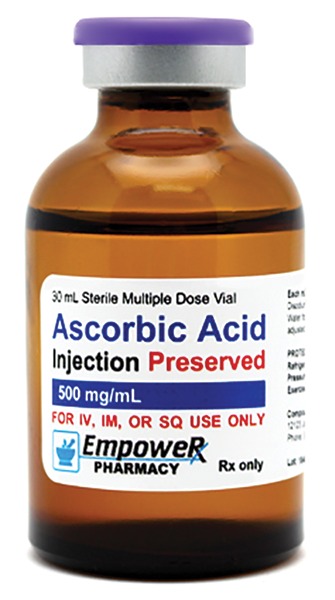
Ascorbic acid

The addition of ascorbic acid to osteoblastic cell lines can stimulate type I collagen production, followed by expression of specific markers associated with osteo-blastic phenotypes, such as alkaline phosphatases (ALP) and osteocalcin. It is also required for *in vitro* mineralized nodule formation of osteoblasts. Ishikawa et al studied the effect of ascorbic acid on PDL cells and observed that ascorbic acid increased the ALP activity, which is required for the binding of PDL cells to type I collagen via 2 beta 1 integrin, whose expression is again increased by ascorbic acid. As type I collagen production is considered to be an initial process in differentiation of PDL cells, it may serve as a potential storage medium (Fig. 13).^2^

## CONCLUSION

An appropriate storage media is recommended for the protection of PDL cells following trauma. Several storage media have been proposed, HBSS being the optimal. All storage media have been shown to lose their clinical effectiveness with time. Gatorade, saline, saliva, contact lens solution, and tap water have been considered non-physiological and are not recommended due to factors, such as pH and osmolarity. Tooth rescue box, ViaSpan, and EM are effective media but are not feasible due to factors, such as cost and lack of availability. Pasteurized milk, egg white, propolis, and coconut water have shown promising results and are readily available. The tooth storage media that are mostly favored when comparing efficacy (in maintaining PDL cell viability) in the literature include: Eagle’s culture medium = ViaSpan = HBSS > milk ≥ propolis ≥ green tea ≥ egg > coconut water. To sum up, HBSS and pasteurized milk are the most appropriate clinically recommended storage media.
